# A Case Report of Potato Allergy With Atypical Manifestations in a 2-Year-Old Child

**DOI:** 10.1155/crpe/2294523

**Published:** 2025-09-07

**Authors:** Nikolaos Kitsos, Lucas Kourentis

**Affiliations:** Department of Pediatrics, University of Thessaly, Larissa, Greece

## Abstract

Food allergies are an increasing global health concern, affecting up to 6%–8% of children and 3%–4% of adults. While common allergens such as milk, eggs, and peanuts are well-documented, rare food allergies, such as those to potatoes, remain underrecognized. Potatoes, a staple food worldwide, can trigger allergic reactions in susceptible individuals due to proteins like patatin (Sol t 1) and protease inhibitors. This case report describes a two-year-old child presenting with recurrent gastrointestinal and respiratory symptoms, ultimately diagnosed with a potato allergy. The child exhibited intermittent vomiting, diarrhea, and wheezing, with episodes temporally linked to potato consumption. Initial clinical evaluations, including hydration and dietary modifications for lactose intolerance or viral gastroenteritis, provided temporary relief but failed to address the underlying cause. A worsening pattern of symptoms, including perioral redness and worsening wheezing, prompted referral to a pediatric allergist. Diagnostic workup revealed elevated eosinophil counts, increased total IgE levels, and significant skin prick test (SPT) reactivity to potato extract. Serum-specific IgE testing confirmed sensitization to Sol t 1. Management included a strict potato-free diet, with additional precautionary exclusion of cross-reactive foods within the Solanaceae family. This intervention led to complete resolution of symptoms, improved weight gain, and enhanced quality of life. Long-term follow-up demonstrated a decline in specific IgE levels, although oral food challenges were deferred due to initial symptom severity. This case underscores the importance of considering rare food allergies in the differential diagnosis of recurrent, nonspecific gastrointestinal and respiratory symptoms in children. Early recognition, aided by targeted allergy testing, can prevent diagnostic delays and unnecessary investigations. Effective management through dietary modifications not only resolves symptoms but also empowers families with the tools to ensure long-term safety and well-being. This report contributes to the growing awareness of potato allergy and its atypical presentations.

## 1. Introduction

Food allergies are growing globally, with an estimated prevalence of 6%–8% in children under the age of five and 3%–4% in adults [1, 2]. While most cases involve well-known allergens such as milk, eggs, peanuts, or tree nuts, rarer allergies to foods like potatoes are often underdiagnosed and underreported [3, 4]. Potatoes, a staple food worldwide, are rarely considered allergenic. However, in susceptible individuals, the allergenic proteins in potatoes, such as the storage protein patatin (Sol t 1) and protease inhibitors, can trigger a spectrum of immune responses ranging from mild skin irritations to severe anaphylactic reactions [5, 6].

Children presenting with food allergies often exhibit recognizable symptoms, such as urticaria, angioedema, or gastrointestinal symptoms, immediately following allergen exposure. These symptoms are easy recognized by the clinician and lead to diagnosis and management. However, in cases where symptoms are delayed or nonspecific—such as chronic wheezing, diarrhea, or failure to thrive—the underlying allergy can remain elusive [7, 8].

Despite the widespread consumption of potatoes, reports of IgE-mediated potato allergy in young children are scarce. Furthermore, the literature rarely addresses presentations dominated by gastrointestinal symptoms rather than immediate-type reactions such as urticaria or anaphylaxis. This case report addresses this knowledge gap by presenting a child with an atypical clinical spectrum. Early recognition of such atypical presentations is crucial to avoid unnecessary investigations, ensure prompt dietary management, and improve patient outcomes [9, 10].

## 2. Case Presentation

### 2.1. Patient Information

The patient, a two-year-old male, was referred to the pediatric clinic with a history of recurrent gastrointestinal and respiratory symptoms over the preceding 3 months. The primary complaints included intermittent vomiting, loose stools, and occasional wheezing. Parents also reported irritability and a general reluctance to eat, particularly during episodes of illness. These symptoms appeared episodic, resolving spontaneously after few days, only to recur without any identifiable triggers. The child had no previous hospitalizations and had been otherwise healthy prior to this presentation. A detailed dietary history revealed that the child consumed a varied diet, including fruits, vegetables, grains, an dairy and meat products. However, upon further questioning, the parents noted a potential link between the onset of symptoms and the consumption of meals containing mashed potatoes. While this correlation was initially dismissed as coincidental, closer observation revealed that episodes of vomiting and diarrhea tended to occur within few hours after consuming dishes containing potatoes. Other members of the household consumed the same meals without any adverse effects [11, 12].

The child had a history of mild eczema as an infant, which improved with regular use of emollients. There was no prior diagnosis of food allergies or sensitivities. The family history was notable for atopic conditions, including asthma in the father and seasonal allergies in the mother. However, there was no known family history of food allergies [13, 14].

### 2.2. Initial Symptoms and Response

The child's symptoms began insidiously, initially presenting as mild irritability, decreased appetite, and loose stools. Over time, the symptoms escalated, with parents observing vomiting, diarrhea, and wheezing. During these episodes, the child exhibited fatigue, pallor, and irritability, prompting the parents to seek medical attention. There were no associated symptoms such as fever, rash, or abdominal pain, which might suggest an infectious etiology. Initial treatment by the primary care physician included hydration, antiemetics, and dietary adjustments to rule out lactose intolerance or viral gastroenteritis. Symptomatic relief was temporary, and symptoms recurred intermittently [15]. As wheezing worsened, a bronchodilator inhaler was prescribed, offering only partial benefit. The condition evolved from mild eczema in infancy to repeated gastrointestinal and respiratory symptoms before diagnosis.  Table 1 outlines the clinical course, management steps, and eventual resolution through dietary intervention.

### 2.3. Exacerbation of Symptoms

During one episode, the parents reported that shortly after eating mashed potatoes, the child developed a rash around his mouth, worsening wheezing, and generalized irritability. The child also exhibited persistent scratching, particularly on his arms, leading to excoriations. Concerned by the worsening pattern, the parents brought the child to the emergency department, where he was evaluated for possible asthma or allergic reactions [16].

### 2.4. Clinical Findings

**Figure 1:**
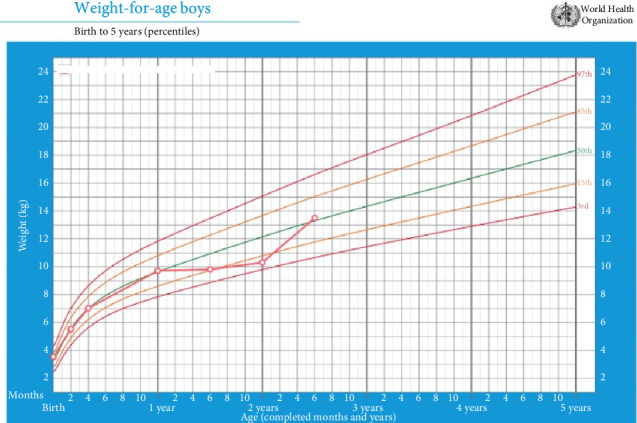
Growth chart of the 2-year-old boy demonstrating improvement in weight-for-age percentile following intervention. Initial weight measured at 10.3 kg (10th percentile); subsequent assessment postintervention, 6 months later, showed weight increased to 13.5 kg, aligning with the 50th percentile for age, indicating significant catch-up growth.

At presentation, the child was alert but appeared fatigued. He weighed 10.3 kg (10th percentile for age), but following dietary intervention, weight improved to 13.5 kg (50th percentile), as illustrated in the growth chart (Figure 1), highlighting significant catch-up growth over six months. Physical examination revealed the following:

• Skin: Erythematous, dry patches over the cheeks, and extensor surfaces of the arms, consistent with eczema. Perioral redness was noted.
• Respiratory System: Mild wheezing was audible in both lung fields, with no signs of acute respiratory distress. Oxygen saturation was 98% on room air.
• Gastrointestinal System: Soft, nondistended abdomen with normal bowel sounds and no tenderness or organomegaly.

Neurological, cardiovascular, and other systemic examinations were unremarkable [17].

### 2.5. Initial Diagnostic Workup

Laboratory investigations were performed to identify potential allergic or infectious etiologies:

1. Complete blood count (CBC): Elevated eosinophil count (9%) (age-specific reference value < 5%), with a normal white blood cell count. This finding raised suspicion for an allergic condition [18].
2. Serum IgE levels: Total IgE levels were elevated at 250 kU/L (age-specific reference value < 60 kU/L), further supporting an atopic background [19].
3. Stool analysis: No evidence of pathogens, parasites, or occult blood, ruling out infectious gastroenteritis [20].

A referral to a pediatric allergist was initiated for further evaluation, given the recurrent nature of the symptoms and the suggestive dietary correlation [21].

### 2.6. Allergy Workup

**Figure 2:**
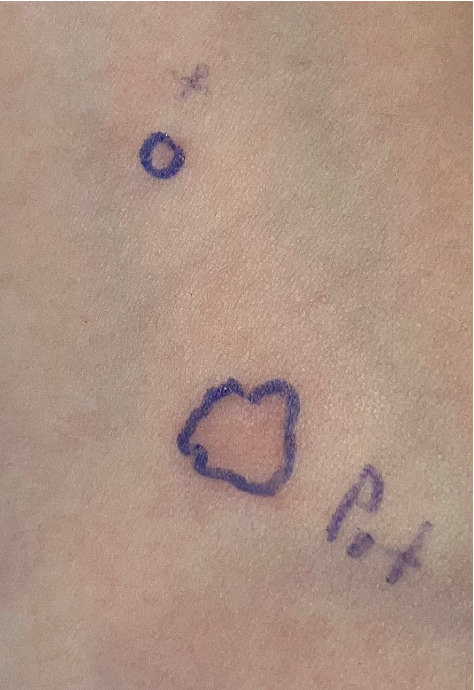
Skin prick test (SPT) results clearly show the significant reaction to potato extract (7 mm), supporting the diagnosis of potato allergy.

The allergist conducted a detailed history and performed skin prick test (SPT) for common food allergens, including milk, egg, wheat, soy, nuts, fish, and potato. SPT revealed a marked reaction to potato extract, producing a 7 mm wheal—more than double the histamine control (3 mm)—as demonstrated in Figure 2, which visually underscores the diagnostic significance of the SPT findings. Other allergens tested negative, ruling out polysensitization [22]. Serum-specific IgE testing for Sol t 1, the major allergenic protein in potatoes, was ordered to confirm sensitization. The results showed elevated-specific IgE levels (2.4 kU/L) to potato proteins, solidifying the diagnosis of potato allergy [23].

### 2.7. Food Elimination Trial

Based on these findings, the child was placed on a strict potato-free diet. All forms of potato, including mashed, fried, and processed potato-based products, were removed from his meals. Given the botanical classification of potatoes within the Solanaceae (nightshade) family, the parents were also advised to eliminate cross-reactive foods such as tomatoes, eggplants, and peppers during the initial elimination phase. These foods share homologous proteins with potatoes, including patatin-like and protease inhibitor proteins, which can sometimes trigger reactions in sensitized individuals. Although cross-reactivity among Solanaceae members is not universally observed, excluding these foods initially can help prevent confounding allergic responses and allow clearer assessment of symptom resolution. After a period of symptom remission, these foods may be cautiously reintroduced under medical supervision to evaluate tolerance and refine long-term dietary restrictions. [24].

Within 2 weeks of dietary modification, the parents reported a marked improvement in the child's symptoms. Vomiting and diarrhea ceased entirely, and wheezing episodes resolved. The child's eczema also improved significantly, requiring only routine emollient use. Over the following month, the child regained his appetite and energy levels, with steady weight gain noted during follow-up visits [25].

### 2.8. Follow-Up and Outcomes

At the 6-month follow-up, repeating allergy testing demonstrated a slight decline in specific IgE levels to potatoes, suggesting possible attenuation of the allergic response. The parents reported no further reactions and expressed confidence in managing the allergy. A structured oral food challenge (OFC) was deferred, given the severity of the initial symptoms and the child's young age [26, 27].

### 2.9. Future Management Strategies and Tolerance Monitoring

Although an OFC was initially deferred because of the severity of the child's symptoms, it remains an essential tool to assess the potential development of tolerance. The observed decline in specific IgE levels to potato at the 6-month follow‐up suggests that the allergic response may be attenuating. In such cases, periodic re‐evaluation—including a carefully supervised oral provocation test under controlled conditions in a clinical setting—may be considered after a longer symptom‐free period to determine if the child has acquired tolerance.

Long‐term management of this patient will involve continued dietary vigilance, with regular follow-ups to monitor immunologic markers such as specific IgE levels and to identify any changes in clinical reactivity. Education of the family on allergen avoidance, recognition of early signs of accidental exposure, and proper use of emergency medications remain a cornerstone of ongoing care.

While immunotherapy has emerged as a potential approach for more common food allergies, its application to potato allergy is not yet well established due to limited evidence and the rarity of the condition. Future research may elucidate whether desensitization protocols could be safely and effectively implemented for such rare allergens. Until then, the management strategy will continue to focus on avoidance, periodic reassessment, and the potential reintroduction of the allergen under medical supervision when indicated.

### 2.10. Unique Features of Presentation

This case was noteworthy for its atypical symptomatology. Unlike classic immediate hypersensitivity reactions to food allergens, which often present with acute urticaria, angioedema, or anaphylaxis, this child exhibited predominantly gastrointestinal and respiratory symptoms. The delayed recognition of potato as the offending allergen underscores the diagnostic challenges posed by rare food allergies with nonclassical presentations [28, 29].

An important differential diagnosis considered was food protein–induced enterocolitis syndrome (FPIES), a non-IgE-mediated food allergy that typically presents with delayed-onset vomiting, diarrhea, and lethargy in infants and young children. The episodic gastrointestinal symptoms without initial cutaneous or respiratory involvement raised this possibility. However, the later emergence of wheezing, eczema, perioral rash, and the strong positive SPT and serum-specific IgE to potato supported an IgE-mediated mechanism, making FPIES less likely in this case.

## 3. Discussion

This case illustrates the complexities of diagnosing and managing rare food allergies in young children. The patient presented with nonclassical features of food allergy, including recurrent vomiting, diarrhea, wheezing, and eczema, without the acute urticaria or anaphylaxis typical of IgE-mediated reactions [30, 31]. This blend of gastrointestinal and respiratory symptoms highlights a potentially mixed mechanism, involving both IgE- and non-IgE-mediated immune responses. Notably, only one previous report in the literature described a co-occurrence of FPIES and immediate-type (IgE-mediated) potato allergy, underscoring the rarity and clinical novelty of this presentation [32].

While the final diagnosis was IgE-mediated potato allergy, the initial presentation with isolated gastrointestinal symptoms and lack of immediate hypersensitivity signs also warranted consideration of FPIES in the differential diagnosis. FPIES is characterized by delayed vomiting and diarrhea several hours after ingestion of the trigger food, often without skin or respiratory involvement. Although potato is a recognized trigger in some FPIES cases, the positive IgE findings and resolution of respiratory and cutaneous symptoms on elimination supported an IgE-mediated rather than a cell-mediated allergic response. This highlights the diagnostic challenge posed by overlapping features of non-IgE- and IgE-mediated food allergies [33].

Compared to earlier cases of potato allergy reported primarily in dermatological or anaphylactic contexts, this case demonstrates a broader symptom spectrum that includes respiratory and gastrointestinal manifestations. Recent studies have identified patatin and other Solanaceae-derived proteins as emerging allergens of concern in pediatric populations [34, 35]. In our case, the identification of Sol t 1-specific IgE provided a clear diagnostic marker, yet the history of delayed-onset gastrointestinal symptoms suggests that IgE sensitization alone does not explain the entire clinical picture. This aligns with newer evidence suggesting that mixed-type allergic responses can occur even within a single exposure framework [36].

Our case both aligns with and diverges from previous literature in meaningful ways. For instance, while Akeson et al. [4] and Jensen-Jarolim & Einhorn [37] documented dermatologic and immediate-onset responses to potato allergens, they did not report systemic, delayed gastrointestinal symptoms as part of the clinical spectrum. Additionally, while the case by Adel-Patient et al. [24] identified cross-reactivity with other Solanaceae family members, such as tomato and eggplant, our proactive dietary exclusion of these foods—followed by subsequent tolerance—offers further clinical insight into management flexibility. This reflects an evolution in practice supported by recent work from Nguyen and Allen [38], who recommend phased reintroduction protocols tailored to individual IgE profiles.

Recent literature [39, 40] has emphasized the diagnostic difficulty of non-IgE mediated allergies in children, where symptoms like vomiting or loose stools may be misattributed to viral or dietary intolerances. This case reinforces the need to maintain a broad differential diagnosis in pediatric patients with persistent, unexplained symptoms. The resolution of symptoms following potato elimination supports the theory that non-IgE-mediated pathways may have contributed, particularly when considering the child's partial response to bronchodilators and recurring eczema flares.

The management approach in this case aligns with current best practices for rare food allergies, emphasizing strict allergen avoidance, precautionary exclusion of cross-reactive foods (e.g., tomato and eggplant), and close follow-up. The 6-month follow-up showing decreased IgE levels is promising, consistent with observations that some children may exhibit tolerance development over time. However, long-term outcomes in children with uncommon food allergies remain insufficiently studied, and more robust cohort studies are needed.

In summary, this case underscores several key themes in pediatric allergy diagnosis and management: the importance of detailed dietary history, integration of targeted diagnostic testing, and patient–family education. It contributes novel insights into the atypical presentation of potato allergy, aligning with current trends in recognizing and managing less common but clinically significant food allergens. By situating this case within the existing body of literature and contemporary developments in pediatric allergy research, we highlight the necessity for sustained clinical vigilance toward rare food allergens and advocate for an integrated diagnostic framework combining immunological, clinical, and dietary assessment to improve prognostic accuracy and therapeutic outcomes.

## Figures and Tables

**Table 1:** Timeline of events.

Date/time period	Event/observation	Management
Infancy	Mild eczema noted.	Resolved with emollients; no prior food allergy diagnosis.
∼3 months before referral	Intermittent vomiting, diarrhea, and occasional wheezing began.	Symptoms were episodic and resolved spontaneously; no specific trigger identified.
Initial evaluation (primary care)	Treatment included hydration, antiemetics, and dietary modifications (ruled out lactose intolerance and viral gastroenteritis).	Temporary symptomatic relief; symptoms recurred intermittently.
Escalation of symptoms	Symptoms worsened: Persistent vomiting, diarrhea, wheezing, fatigue, irritability.	Temporarily managed with bronchodilator inhaler.
Shortly after potato consumption	Parents noticed a pattern of symptoms (vomiting, diarrhea, perioral rash, worsening wheezing).	Rash and wheezing prompted emergency department evaluation.
Emergency department visit	Exam: Mild wheezing, perioral redness, fatigue. Lab tests: Elevated eosinophil count (9%), high IgE (250 kU/L).	Findings raised suspicion of allergy. Stool analysis ruled out infections. Referred to pediatric allergist.
Allergy evaluation	Skin prick test revealed strong reaction to potato extract (7 mm wheal). Serum IgE confirmed sensitization to Sol t 1.	Diagnosis of potato allergy established.
Dietary intervention	Strict potato-free diet initiated. Cross-reactive foods (e.g., tomatoes, eggplants) excluded initially.	Within 2 weeks, vomiting, diarrhea, wheezing resolved; eczema improved.
Follow-up at 1 month	Symptom-free. Weight gain and appetite improved.	Parents confident in managing dietary restrictions.
Follow-up at 6 months	Repeat testing showed slight decline in specific IgE levels to potato.	Oral food challenge deferred due to initial symptom severity.
Long-term outcome	No recurrence of symptoms; parents reported high quality of life.	Educational focus on allergen avoidance and cross-reactivity management proved effective.

## Data Availability

The data that support the findings of this study are available on request from the corresponding author. The data are not publicly available due to privacy or ethical restrictions.
