# Correction to “A Case Report of Potato Allergy With Atypical Manifestations in a 2‐Year‐Old Child”

**DOI:** 10.1155/crpe/9764947

**Published:** 2026-02-04

N. Kitsos and L. Kourentis, “A Case Report of Potato Allergy With Atypical Manifestations in a 2‐Year‐Old Child,” *Case Reports in Pediatrics*, vol. 2025 (2025), https://doi.org/10.1155/crpe/2294523.

In the article titled “A Case Report of Potato Allergy With Atypical Manifestations in a 2‐Year‐Old Child”, Dr. Aspasia Michoula and Dr. Ioanna Grivea were missing from the author list.

Dr. Aspasia Michoula and Dr. Ioanna Grivea contributed to the preparation of the manuscript and data analysis in addition to revising the manuscript during peer review.

The corrected author list should read as follows:

[“Nikolaos Kitsos, Aspasia Michoula, Ioanna Grivea, Lucas Kourentis *Department of Pediatrics, University of Tessaly, Larissa, Greece*”]

All authors agree with the abovementioned changes.

We apologize for this error.

